# Cerebral autoregulation: every step counts

**DOI:** 10.1186/s13054-023-04595-3

**Published:** 2023-08-08

**Authors:** Timothée Ayasse, Jacques Duranteau, Anatole Harrois, Jonas Pochard

**Affiliations:** 1 Department of Anaesthesia and Critical Care, AP-HP, Hôpital Kremlin Bicêtre, 94276 Le Kremlin Bicêtre, France; 2 Inserm, UMRS 999, Université Paris Saclay, 94276 Le Kremlin Bicêtre, France

Dear editor,

The study of cerebral autoregulation in real time is one of the most promising development in the management of critically ill patients with acute brain injury. Beyond the single value of optimal cerebral perfusion pressure (CPP), assessing the complete interval of CPP at which cerebral autoregulation is effective, including upper limit of autoregulation (ULA) and lower limit of autoregulation (LLA), could help to personalize hemodynamic objectives. For this reason, we read with great interest the study by Haqiri et al. [1] recently published in Critical Care journal reporting the feasibility of a multiwindow-based algorithm for calculating the PRx-derived LLA named lower limit reactivity (LLR). We are also firmly convinced that deviation of CPP below LLR is associated with prognosis. However, the methods used for the assessment of cerebral autoregulation and the determination of LLR in this article may be questionable for several reasons.

First, as acknowledged by the authors, PRx is derived from spontaneous variations in mean arterial pressure (MAP) and calculated as a moving correlation coefficient between 10 s averages of intracranial pressure (ICP) and MAP waveforms within a 5 min window [2]. This approach requires sufficient variability in MAP over time, which can be limited in the intensive care unit due to meticulous patient monitoring leading to accurate CPP targeting. Consequently, significant variations in MAP are primarily triggered by external factors or happened in response to primary variations in ICP (i.e., pain, tracheal suctioning, position change, change in brain oxygen demand…). This could be acting as confounding variables, akin to noise. Integrating these stimuli into the analysis of cerebral autoregulation is complex as they can induce variations in cerebral blood flow (CBF) unrelated to changes in MAP. This results in a multivariate variability in CBF, making the correlation between MAP and CBF [3] difficult to assess accurately.

Second, the PRx method assesses dynamic cerebral autoregulation, which evaluates the cerebrovascular system’s ability to buffer relative changes in CBF in response to rapid changes in MAP. The cerebrovascular bed behaves as a time-dependent buffer against fluctuations in CBF [4] and is unable to maintain it entirely constant during prompt variations in MAP, even in healthy subjects with intact steady-state cerebral autoregulation [5]. Hence, differentiating between physiological and pathological responses becomes challenging, limiting the PRx method’s ability to provide a comprehensive understanding of cerebral autoregulation. In these scenarios, uncompensated abrupt variations in MAP can significantly impact the interpretation of cerebral autoregulation, even though an appropriate vascular response to a progressive increase in MAP secondary to another trigger may remain intact. Therefore, it questions the clinical significance of including episodes exceeding the cerebrovascular capacity to counterbalance sudden shifts in MAP, and its subsequent impact on the evaluation of cerebral autoregulation.

Third, instead of assessing cerebral autoregulation status at an individual level, studies conducted on PRx have predominantly focused on correlating PRx values and global prognosis [6]. Mathematically, positive PRx values indicate impaired cerebral autoregulation, while negative values mean effective autoregulation. However, no specific threshold has been individually validated for this purpose. In this study, the authors utilize absolute PRx values to characterize the individual status of cerebral autoregulation, based on an index that has been validated through retrospective studies involving heterogeneous populations. As pointed out by the authors, this approach carries the risk of erroneous interpretation of patient’s autoregulatory status. To illustrate, the cutoff value of 0.2 can be reached due to reasons unrelated to LLR, such as the effect of exogenous noise that influence CBF without changes in MAP including variations in partial pressure of arterial carbon dioxide or in brain metabolism.

In our opinion and clinical practice, the study of cerebral autoregulation should be based on simultaneous recording of fluctuations in an output, which is CBF or at least a surrogate of CBF, following induced variations in an input, MAP, that should be consistently of the same nature, the latter still being a matter of debate [7]. This method allows the control of both amplitude of MAP variations and confounding factors influencing the relationship between MAP and CBF [8].

## Data Availability

Not applicable.

## Abbreviations

CBF: Cerebral blood flow
CPP: Cerebral perfusion pressure
ICP: Intracranial pressure
LLA: Lower limit of autoregulation
LLR: Lower limit reactivity
MAP: Mean arterial pressure
PRx: Pressure reactivity index
ULA: Upper limit of autoregulation
